# An Account of the Late Dr. Hugh Martin's Cancer Powder; with Brief Observations on Cancers

**Published:** 1787

**Authors:** Benjamin Rush

**Affiliations:** Professor of Chemistry in the University of Pennsylvania


XII. *An Account of the late Dr. Hugh Martin's Cancer Powder; with brief Observations on Cancers. By* Benjamin Rufh, *M. D., Profeffor of Chemiftry in the Univerfity of Pennfylvania.*—From the *Tranfactions of the American Philofophical Society, held at Philadelphia, for promoting ufeful Knowledge,* Vol. II. 4to. Philadelphia, 1786.

A FEW years ago a certain Dr. Hugh Martin, a furgeon of one of the Pennfylvania regiments ftationed at Fort Pitt during the latter part of the late war, came to this city, and advertifed to cure cancers with a medicine which he faid he had difcovered in the woods in the neighbourhood of the garrifon. As Dr. Martin had once been a pupil of mine, I took the liberty of waiting upon him, and afked him fome queftions refpecting his difcovery. His anfwers were calculated to make me believe that his medicine was of a vegetable nature, and that it was originally an Indian remedy. He fhewed me fome of the medicine, which appeared to be the powder of a well-dried root of fome kind. Anxious to fee the fuccefs of this medicine in cancerous fores, I prevailed upon the Doctor to admit me to fee him apply it in two or three cafes. I obferved in fome inftances he applied a powder to the parts affected, and in others only touched them with a feather dipped in a liquid which had a white fediment, and which he made me believe was the vegetable root diffufed in water. It gave me great pleafure to witnefs the efficacy of the Doctor's applications. In feveral cancerous ulcers, the cures he performed were complete. Where the cancers were much connected with the lymphatic fyftem, or accompanied with a fcrophulous habit of body, his medicine always failed, and in fome inftances did evident mifchief.

Anxious to difcover a medicine that promifed relief in even a few cafes of cancers, and fuppofing that all the cauftic vegetables were nearly alike, I applied the phytolacca or poke root, the ftramonium, the arum, and one or two others, to foul ulcers, in hopes of feeing the fame effects from them which I had feen from Dr. Martin's powder; but in thefe I was difappointed. They gave fome pain, but performed no cures. At length I was furnifhed, by a gentleman from Fort Pitt, with a powder which I had no doubt, from a variety of circumftances, was of the fame kind as that ufed by Dr. Martin.

tin. I applied it to a fungous ulcer, but without producing the degrees of pain, inflammation, or difcharge, which I had been accuftomed to fee from the application of Dr. Martin's powder. After this, I fhould have fufpected that the powder was not a fimple root, had not the Doctor continued upon all occafions to affure me that it was wholly a vegetable preparation.

In the beginning of the year 1784 the Doctor died, and it was generally believed that his medicine had died with him. A few weeks after his death I procured from Mr. Thomas Lieper, one of his adminiftrators, a few ounces of the Doctor's powder, partly with a view of applying it to a cancerous fore which then offered, and partly with a view of examining it more minutely than I had been able to do during the Doctor's life. Upon throwing the powder, which was of a brown colour, upon a piece of white paper, I perceived diftinctly a number of white particles fcattered through it. I fufpected at firft that they were corrofive fublimate; but the ufual tefts of that metallic falt foon convinced me that I was miftaken. Recollecting that arfenic was the bafis of moft of the celebrated cancer powders that have been used in the world, I had recourse to the tests for detecting it. Upon sprinkling a small quantity of the powder upon some coals of fire, it emitted the garlic smell so perceptibly as to be known by several persons whom I called into the room where I made the experiment, and who knew nothing of the object of my inquiries. After this, with some difficulty I picked out about three or four grains of the white powder, and bound them between two pieces of copper, which I threw into the fire. After the copper pieces became red hot, I took them out of the fire, and when they had cooled, discovered an evident whiteness imparted to both of them. One of the pieces afterwards looked like dull silver. These two tests have generally been thought sufficient to distinguish the presence of arsenic in any bodies; but I made use of a third, which has lately been communicated to the world by Mr. Bergman, and which is supposed to be in all cases infallible.

I infused a small quantity of the powder in a solution of a vegetable alkali in water for a few hours, and then poured it upon a solution of blue vitriol in water. The colour of the vitriol was immediately changed to a beautiful green, and afterwards precipitated.

I shall

I ſhall cloſe this paper with a few remarks upon this powder, and upon the cure of cancers and foul ulcers of all kinds.

1. The uſe of cauſtics in cancers and foul ulcers is very ancient, and univerſal; but I believe arſenic to be the moſt efficacious of any that has ever been uſed. It is the baſis of Plunket's and Guy's well-known cancer powders. The great art of applying it ſucceſsfully is to dilute and mix it in ſuch a manner as to mitigate the violence of its action. Dr. Martin's compoſition was happily calculated for this purpoſe. It gave leſs pain than the common or lunar cauſtic. It excited a moderate inflammation, which ſeparated the morbid from the ſound parts, and promoted a plentiful afflux of humours to the ſore during its application. It ſeldom produced an eſcar: hence it inſinuated itſelf into the deepeſt receſſes of the cancers, and frequently ſeparated thoſe fibres in an unbroken ſtate, which are generally called the roots of the cancer. Upon this account, I think, in an ulcerated cancer it is to be preferred to the knife. It has no action upon the ſound ſkin. This Dr. Hall proved by confining a ſmall quantity of it upon his arm for many hours. In thoſe caſes where Dr. Martin uſed ufed it to extract cancerous or fchirrous tumours that were not ulcerated, I have reafon to believe that he always broke the fkin with Spanifh flies.

2. The arfenic ufed by the Doctor was the pure white arfenic. I fhould fuppofe, from the examination I made of the powder with the eye, that the proportion of arfenic to the vegetable powder could not be more than one-fortieth part of the whole compound. I have reafon to think that the Doctor employed different vegetable fubftances at different times. The vegetable matter with which the arfenic was combined in the powder which I ufed in my experiments, was probably nothing more than the powder of the root and berries of the folanum lethale, or deadly nightfhade. As the principal, and perhaps the only, defign of the vegetable addition was to blunt the activity of the arfenic, I fhould fuppofe that the fame proportion of common wheat flour as the Doctor ufed of his cauftic vegetables would anfwer nearly the fame purpofe. In thofe cafes where the Doctor applied a feather dipped in a liquid to the fore of his patient, I have no doubt but his phial contained nothing but a weak folution of arfenic in water. This is no new method of applying plying arfenic to foul ulcers. Dr. Way, of Wilmington, has fpoken in the higheft terms to me of a wafh for foulneffes on the fkin, as well as old ulcers, prepared by boiling an ounce of white arfenic in two quarts of water to three pints, and applying it once or twice a day.

3. I mentioned formerly, that Dr. Martin was often unfuccefsful in the application of his powder. This was occafioned by his ufing it indifcriminately in all cafes. In fchirrous and cancerous tumours, the knife fhould always be preferred to the cauftic. In cancerous ulcers, attended with a fcrophulous or bad habit of body, fuch particularly as have their feat in the neck, in the breafts of females, and in the axillary glands, it can only protract the patient's mifery. Moft of the cancerous fores cured by Dr. Martin were feated on the nofe, or cheeks, or upon the furface or extremities of the body. It remains yet to difcover a cure for cancers that taint the fluids, or infect the whole lymphatic fyftem. This cure I apprehend muft be fought for in diet, or in the long ufe of fome internal medicine.

To pronounce a difeafe incurable, is often to render it fo. The intermitting fever, if left to itfelf, would probably prove frequently, and perperhaps more ſpeedily fatal than cancers. And as cancerous tumours and ſores are often neglected, or treated improperly by injudicious people, from an apprehenſion that they are incurable, (to which the frequent advice of phyſicians, " to let them alone," has no doubt contributed) perhaps the introduction of arſenic into regular practice, as a remedy for cancers, may invite to a more early application to phyſicians, and thereby prevent the deplorable caſes that have been mentioned, which are often rendered ſo by delay, or unſkilful management.

4. It is not in cancerous ſores only that Dr. Martin's powder has been found to do ſervice. In ſores of all kinds, and from a variety of cauſes, where they have been attended with fungous fleſh or callous edges, I have uſed the doctor's powder with advantage.

I flatter myſelf I ſhall be excuſed in giving this detail of a quack medicine, when the ſociety reflect, that it is from the invention and temerity of quacks, phyſicians have derived ſome of their moſt active and uſeful medicines.

XIII. *The*

